# miR-10a in Peripheral Blood Mononuclear Cells Is a Biomarker for Sepsis and Has Anti-Inflammatory Function

**DOI:** 10.1155/2020/4370983

**Published:** 2020-01-22

**Authors:** Guoping Zheng, Guanguan Qiu, Menghua Ge, Jianbiao Meng, Geng Zhang, Jiangmei Wang, Ruoqiong Huang, Qiang Shu, Jianguo Xu

**Affiliations:** ^1^Shaoxing Second Hospital, 123 Yanan Road, Shaoxing, Zhejiang 312000, China; ^2^Tongde Hospital of Zhejiang, 132 Tianmushan Road, Hangzhou, Zhejiang 310007, China; ^3^Children's Hospital, Zhejiang University School of Medicine, National Clinical Research Center for Child Health, 3333 Binsheng Road, Hangzhou, Zhejiang 310052, China

## Abstract

**Background:**

Recent literature has reported the use of circulating microRNAs (miRNAs) as biomarkers for sepsis. Immune cells play an essential role in the pathophysiology of sepsis. The aim of this prospective study was to identify miRNAs in peripheral blood mononuclear cells (PBMC) that could differentiate between sepsis and infection based on Sepsis-3 definition.

**Methods:**

A total of 62 patients (41 with sepsis and 21 with infection suffering from pneumonia but without sepsis) and 20 healthy controls were enrolled into the study. PBMC at admission were examined for a panel of 4 miRNAs (miR-10a, miR-17, miR-27a, and miR-125b), which have been documented to participate in inflammatory response in immune cells, via qRT-PCR. Data were validated in a mouse model of sepsis induced via cecal ligation and puncture (CLP) and THP-1 monocytes.

**Results:**

miR-10a levels in PBMC at admission were significantly lower in sepsis patients compared with patients with infection and healthy controls. miR-10a levels were negatively correlated with disease severity scores as well as levels for c-reactive protein and procalcitonin. In addition, low miR-10a expression had a diagnostic value for sepsis and a prognostic value for 28-day mortality in receiving operating characteristic analysis. Compared with infection patients and healthy controls, PBMC from sepsis patients also had higher levels of mitogen-activated kinase kinase kinase 7 (MAP3K7), a known target protein of miR-10a and an activator of the NF-*κ*B pathway. In the mouse model of CLP-induced sepsis, miR-10a levels in PBMC were significantly decreased as early as 8 h after CLP. Overexpression of miR-10a in THP-1 cells significantly reduced the expression of MAP3K7 and proinflammatory cytokines including IL-6, TNF-*α*, and MCP-1.

**Conclusions:**

PBMC miR-10a levels are decreased in sepsis and negatively correlated with the disease severity. Levels of miR-10a could distinguish between sepsis and infection and predict 28-day mortality. miR-10a plays an anti-inflammatory role in the pathogenesis of sepsis.

## 1. Background

Sepsis is currently defined as a life-threatening organ dysfunction caused by an infection with an increase in the Sequential Organ Failure Assessment (SOFA) score ≥ 2 (Sepsis-3). It is the result of a dysregulated immune response to infection and one of the leading causes of death in intensive care units [1]. It is estimated that about 30 million patients worldwide are afflicted with sepsis, with potentially over 5 million deaths annually [2]. During the initial phase of sepsis, activated monocytes/macrophages and neutrophils release a variety of proinflammatory cytokines. The hyperinflammatory response and secondary immunosuppression often lead to multiple organ failure, the major cause of mortality [3].

Early and differential diagnosis of sepsis is essential to decrease morbidity and mortality through prompt antibiotics, vasopressors, and adjuvant therapy [4]. Existing biomarkers for sepsis, such as c-reactive protein (CRP), procalcitonin (PCT), and IL-6, have limited specificity and sensitivity. MicroRNAs (miRNAs) may also serve as diagnostic and prognostic biomarkers in sepsis. miRNAs are short noncoding 18- to 25-nucleotide RNAs that bind to the complementary sequence of target mRNAs, mostly in the 3′ untranslated region. They negatively regulate protein expression of target genes via translational repression or mRNA degradation [5]. It has been estimated that up to 30% of all human genes are miRNA targets, with some miRNAs targeting large numbers of mRNAs [6, 7]. Accumulating evidences have demonstrated that miRNAs are regulators of immune response [8]. miRNA-146a and miRNA-146b were shown to target at TRAF6 and IRAK1 and regulated apoptosis of dendritic cells and cytokine production [9]. miR-223 levels were elevated in T cells of patients with rheumatoid arthritis [10]. In addition, miR-155 promoted the survival of *Mycobacterium tuberculosis*-infected macrophages and favored the survival and function of T cell immune response [11].

Circulating miRNAs, such as miR-15a, miR-25, miR-34a, miR-133a, miR-146, miR-150, and miR-223, have been described as the biomarkers for sepsis [12–15]. However, the circulating miRNAs are a mixture of miRNAs released extracellularly or contained at apoptotic bodies and exosomes [16]. They may not be ideal biomarkers for early diagnosis. The main sources of inflammatory mediators in sepsis are immune cells. Recently, microRNAs such as miR-10a, miR-17, miR-27a, and miR-125b have been shown to modulate inflammatory response in immune cells. For example, miR-10a was decreased in peripheral blood mononuclear cells (PBMC) from patients of inflammatory bowel diseases and upregulated after treatment with monoclonal antibody for antitumor necrosis factor. miR-10a also inhibited the expression of IL-12/IL-23p40 in human dendritic cells and blocked the Th1/Th17 cell immune response [17]. Lipopolysaccharide (LPS) activated alveolar macrophages by increasing the expression of miR-17, which targeted signal-regulatory protein *α* and elevated macrophage infiltration, phagocytosis, and secretion of proinflammatory cytokines [18]. In addition, LPS downregulated miR-27a expression in macrophages as a regulatory mechanism to block overly exuberant inflammatory response [19]. In patients with rheumatoid arthritis, miR-125b expression in CD14^+^ blood monocytes was reduced as compared with healthy controls and negatively correlated with the expression of its target proteins BIK and MTP18, resulting in resistance to oxidative stress and apoptosis [20].

We hypothesized that expression of miRNAs is altered in immune cells during sepsis and might serve as early biomarkers for sepsis. Levels of miR-10a, miR-17, miR-27a, and miR-125b in PBMC were compared among sepsis, infection, and control groups. The results revealed a downregulation of miR-10a in sepsis patients. Downregulation of miR-10a was further validated in a mouse model of cecal ligation and puncture- (CLP-) induced sepsis. Functional experiments in vitro identified an anti-inflammatory role of miR-10a.

## 2. Methods

### 2.1. Study Subjects

Between January 2018 and May 2018, a total of 41 sepsis patients hospitalized in the ICU of Shaoxing Second Hospital were consecutively enrolled in the study. All sepsis patients fulfilled the definition of the Third International Consensus Definitions for Sepsis and Septic Shock (Sepsis-3, infection + increase ≥ 2 in SOFA score) [1]. Twenty-one patients admitted in the ICU for infection (infection+≥2 criteria for systemic inflammatory response syndrome), as a result of pneumonia but without sepsis according to Sepsis-3, were also enrolled. Eligible patients were at least 18 years of age and diagnosed with sepsis or infection without sepsis within the previous 24 h at any time during their stay in the ICU. Blood samples were collected within 24 hours of admission to the ICU. Twenty healthy volunteers without any signs of infection were enrolled as control (Table 1). Exclusion criteria included cancer, chronic inflammatory diseases, brain injury, HBV/HCV/HIV infection, pregnancy, and refusal of consent. The study was carried out in compliance with the Declaration of Helsinki and was preapproved by the ethics committee of Shaoxing Second Hospital. Written informed consents were obtained from all enrolled patients and healthy volunteers. Demographic and clinical data for infection and septic patients were extracted from the electronic medical record. Patients were observed from ICU admission to day 28 of hospital stay or death.

### 2.2. Mouse Model of Cecal Ligation and Puncture (CLP)

All studies used C57BL/6 male mice (6-8 weeks old; Shanghai Laboratory Animal Center, Shanghai, China). Animal protocols were approved by the animal care committee at Zhejiang University School of Medicine and were in compliance with institutional guidelines. Mice were anesthetized with 4% trichloroacetaldehyde via intraperitoneal injection. A 2 cm ventral midline incision was performed to allow the exposure of cecum. The cecum was ligated 1 cm from the apex with 3-0 silk suture, punctured twice with a 22-gauge needle, and extruded a small amount of fecal material. The abdominal incision was sutured in two layers. Sham surgery was performed with only cecum exposure without ligation and puncture of the cecum in control animals. Isotonic saline solution (1 ml/mouse) was administered subcutaneously to all mice to prevent dehydration. Mice were sacrificed at 8 and 24 h after initial sham or CLP procedure to collect blood for analysis.

### 2.3. Total RNA Extraction from Peripheral Blood Mononuclear Cells

PBMC were separated by density gradient centrifugation using blood samples collected in heparinized tubes. Briefly, blood samples were mixed with an equal volume of phosphate-buffered saline. The mixture was loaded on the top of a Ficoll-Paque density gradient (Cedarlane, Burlington, NC, USA) and pelleted at 2200 rpm for 20 min. PBMC at the interface were harvested and washed twice with phosphate-buffered saline.

### 2.4. Culture and Treatment of THP-1 Monocytes

THP-1 cells were obtained from the ATCC (Manassas, VA) and maintained in RPMI 1640 medium (Thermo Fisher Scientific) supplemented with 10 U/ml penicillin-streptomycin, 2 mM L-glutamine, and 10% fetal bovine serum (Thermo Fisher Scientific) at 37°C with 5% CO_2_. Cells in log-phase were used for the experiments. THP-1 cells were treated with LPS (100 ng/ml, *E. coli* LPS 055:B5, Sigma-Aldrich) for 24 h. After the treatment, cells were harvested for qRT-PCR analysis.

### 2.5. Real-Time Quantitative Reverse Transcriptase Polymerase Chain Reaction (qRT-PCR) and miRNA-Specific Primers

Total RNA was isolated from cells using the TRIzol Reagent (Thermo Fisher Scientific). For mRNA analysis, reverse transcription reaction was performed via the PrimeScript™ RT Reagent Kit (Takara Bio, Kusatsu, Japan) according to the manufacturer's instructions. qRT-PCR was performed using SYBR Green™ Premix Ex Taq™ (Takara Bio) on the LightCycler 480 II (Roche). miRNA was determined using the Mix-X™ miRNA First Strand Synthesis Kit (Takara Bio) followed by the LightCycler 480 II via the Mir-X miRNA qRT-PCR SYBR Kit (Takara Bio). All target genes were standardized to *β*-actin for mRNA or U6 snRNA for miRNA using the standard *ΔΔ*Ct method. The primer sequences used are listed as follows: IL-6 forward 5′ACTCACCTCTTCAGAACGAATTG3′, reverse 5′CCATCTTTGGAAGGTTCAGGTTG3′; TNF-*α* forward 5′CAGGCGGTGCCTATGTCTC3′, reverse 5′CGATCACCCCGAAGTTCAGTAG3′; MCP-1 forward 5′CAGCCAGATGCAATCAATGCC3′, reverse 5′TGGAATCCTGAACCCACTTCT3′; *β*-actin forward 5′CGTTGACATCCGTAAAGACC3′, reverse 5′AACAGTCCGCCTAGAAGCAC3′; miR-10a 5′CTGTAGATCCGAATTTGTGA3′; miR-27a 5′AGTGGCTAAGTTCCGCAA3′; miR-125b 5′CCCTGAGACCCTAACTTGTGAAA3′; miR-17 5′GTGCTTACAGTGCAGGTAGAA3′; and U6 5′TCGTGAAGCGTTCCATATTTTTAA3′.

### 2.6. Transducing miR-10a into THP-1 Cells

Mouse-miR-10a-5p and control lentivirus were purchased from Genechem (Shanghai, China). THP-1 cells were infected with lentiviruses (multiplicity of infection of 20) and cultured for 4 days. THP-1 cells were then stimulated with LPS (100 ng/ml) for 24 hours. After that, the cells were harvested for qRT-PCR and Western blot analyses.

### 2.7. Western Blot Analysis

Western blot analysis was performed as described previously [21]. Protein samples were extracted from PBMC of control, infection, and sepsis groups at admission. Equal amounts of protein were loaded on SDS-PAGE and transferred to a nitrocellulose membrane. The membrane was probed with a rabbit anti-human mitogen-activated kinase kinase kinase 7 (MAP3K7; TAK1) primary antibody (Cell Signaling Technology, Danvers, MA), followed by incubation with a peroxidase-conjugated goat anti-rabbit secondary antibody (MultiSciences, Hangzhou, China), and detected via enzyme-linked chemiluminescence using the EZ-ECL Kit (Biological Industries, Kibbutz Beit-Haemek, Israel). MAP3K7 levels were normalized to GAPDH expression levels.

### 2.8. Statistical Analysis

All continuous values with normal distribution were presented as mean ± standard error of the mean (SEM) and analyzed via Student's *t*-test or an analysis of variance (ANOVA), followed by the Dunn post hoc test for multiple groups. All continuous variables with skewed distribution were expressed as median (interquartile range (IQR)) and analyzed via the Whitney *U* test. Receiver operating characteristic (ROC) curves and the area under the ROC curves (AUC) were performed to determine the diagnostic and prognostic values of miR-10a. Statistical analysis was carried out using the GraphPad Software (Prism 5.01, GraphPad Software) and SPSS 22.0. Differences were considered as statistically significant if *p* < 0.05.

## 3. Results

### 3.1. Study Patient Characteristics

Twenty-one patients with infection, 41 patients with sepsis, and 20 healthy controls were included in the study. The demographic and clinical characteristics of the study patients are shown in Table 1. The major etiologies were pneumonia for both infection and sepsis patients. Sepsis patients had a significantly higher SOFA score and higher CRP and PCT levels than infection patients at enrollment. Patients with infection and sepsis were slightly older than healthy controls, but there was no statistically significant difference in age among the three groups (*p* > 0.05).

### 3.2. PBMC miR-10a Levels Are Reduced in Sepsis Patients

**Figure 1:**
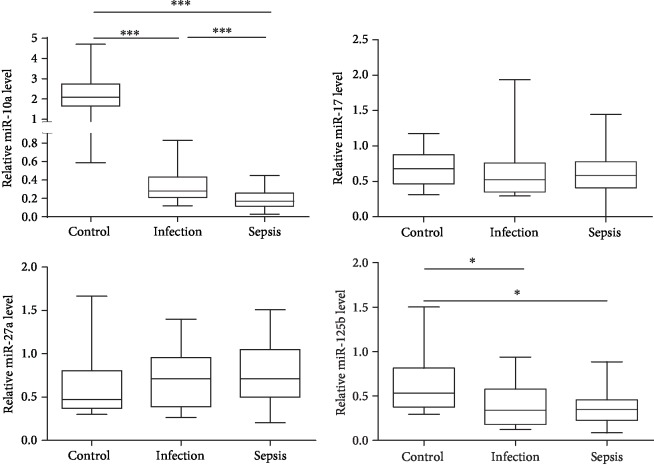
PBMC miR-10 levels in sepsis. The box and whisker plots represent the levels of miR-10a, miR-17, miR-27a, and miR-125b in PBMC of healthy controls (*n* = 20), infection patients (*n* = 21), and sepsis patients (*n* = 41). The plot shows the median (lines within boxes), interquartile range (bounds of boxes), and error bars (upper and lower ranges). ^∗^*p* < 0.05 and ^∗∗∗^*p* < 0.001.

To explore the potential value of PBMC miRNAs as a biomarker for sepsis, levels of 4 inflammation-related miRNAs (miR-10a, miR-17, miR-27a, and miR-125b) in PBMC at admission were examined in 41 patients with sepsis, 21 patients with infection, and 20 healthy controls via qRT-PCR (Figure 1). miR-10a levels in sepsis patients were lower than those in infection patients and healthy controls. Infection patients also showed reduced levels of miR-10a compared with healthy controls. There was no difference in miR-17 and miR-27a levels among the control, infection, and sepsis patients. miR-125b expression was decreased in sepsis and infection patients compared with healthy controls. However, there was no difference in miR-125b expression between sepsis and infection patients, indicating that miR-125b could not differentiate sepsis from infection.

### 3.3. miR-10a Levels in PBMC from Sepsis Patients Correlate with Disease Severity Scores and Existing Biomarkers for Sepsis

**Figure 2:**
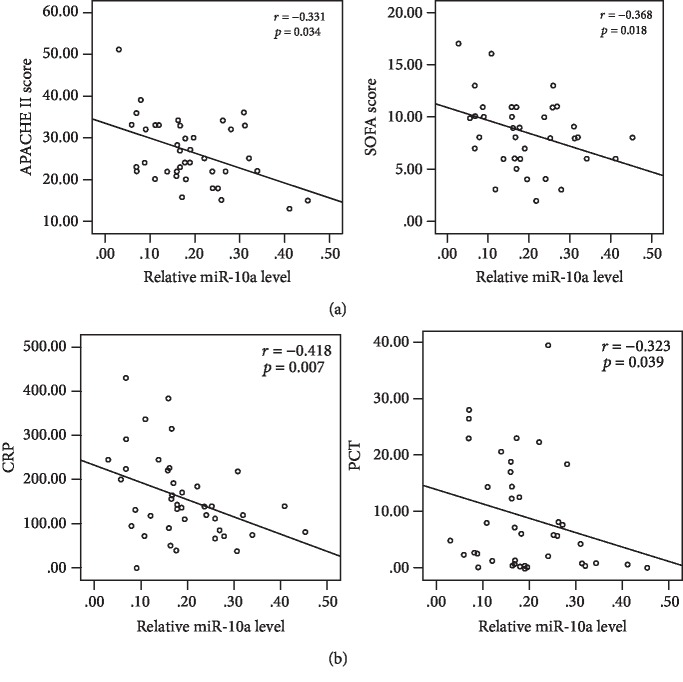
Correlation analysis of PBMC miR-10a levels with disease severity scores and sepsis biomarkers. (a) The curve was plotted by miR-10a relative expression values at admission of 41 sepsis patients to their respective APACHE II and SOFA scores. (b) The curve was plotted by miR-10a relative expression levels at admission to their respective CRP and PCT values. Each circle represents an individual patient.

To elucidate the clinical relevance of miR-10a in sepsis, Spearman's correlation analysis was conducted between levels of miR-10a in PBMC and the disease severity scoring systems using the Acute Physiology and Chronic Evaluation II (APACHE II) and the SOFA. Strong negative linear relationships existed between levels of miR-10a and APACHE II scores (*r* = −0.331, *p* = 0.034) as well as SOFA scores (*r* = −0.368, *p* = 0.018) (Figure 2(a)). In addition, miR-10a levels in PBMC were also significantly correlated to levels of CRP (*r* = −0.418, *p* = 0.007) and PCT (*r* = −0.323, *p* = 0.039), two popular biomarkers of sepsis (Figure 2(b)).

### 3.4. PBMC miR-10a Has Diagnostic Performance for Sepsis

**Figure 3:**
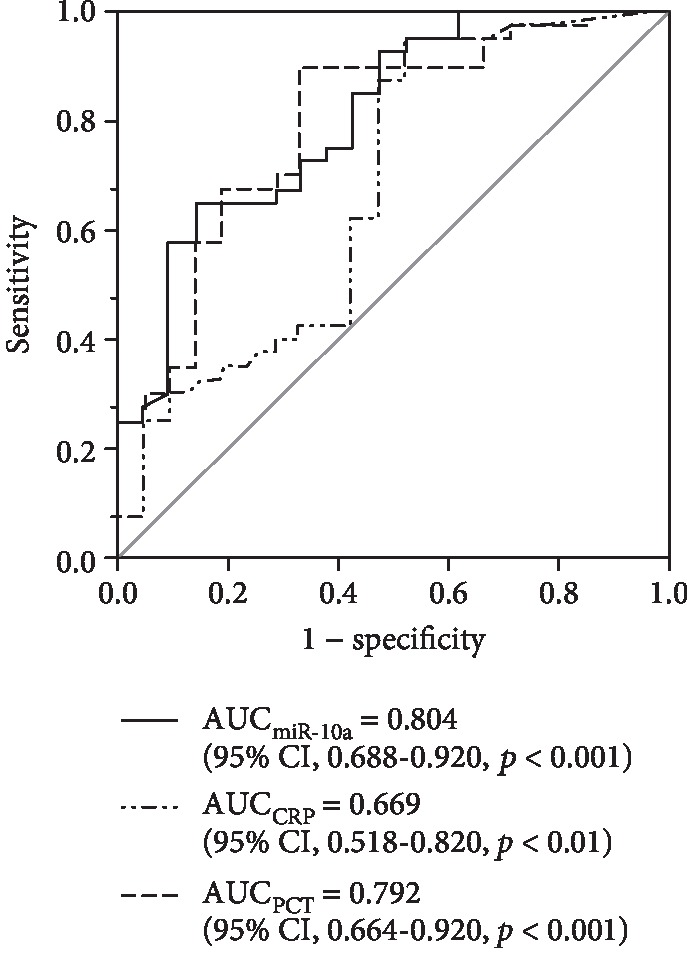
ROC curves for the prediction of sepsis. ROC curves present sensitivity and specificity of PBMC miR-10a, CRP, and PCT for predicting sepsis.

ROC curve analysis was applied to compare the diagnostic value of PBMC miR-10a, CRP, and PCT between infection and sepsis. The AUC for miR-10a (AUC = 0.804; 95% CI, 0.688-0.920; *p* < 0.001) was comparable to that of PCT (AUC = 0.792; 95% CI, 0.664-0.920; *p* < 0.001) and better than the diagnostic performance of CRP (AUC = 0.669; 95% CI, 0.518-0.820; *p* < 0.01) (Figure 3). These findings suggest that PBMC miR-10a could distinguish between sepsis and infection and could perform at least as well as PCT and CRP for sepsis diagnosis. When the cut-off point for miR-10a was set at 0.18, it had a sensitivity of 65.0% and a specificity of 85.7%.

### 3.5. PBMC miR-10a Has Predictive Value for 28-Day Mortality

**Figure 4:**
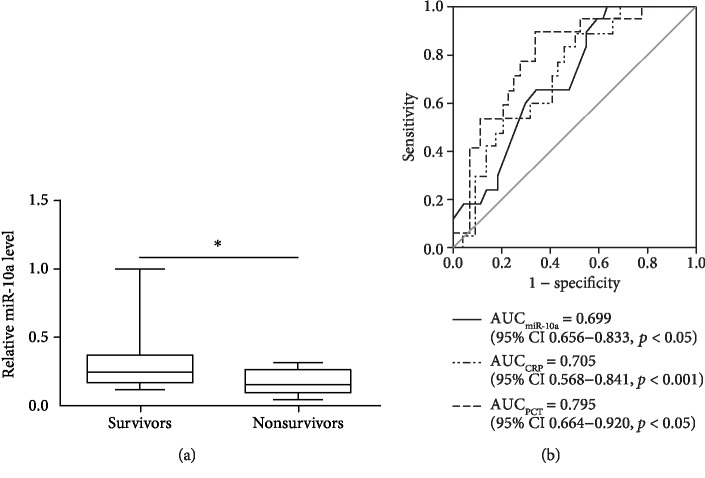
ROC curves for the prediction of 28-day mortality. (a) PBMC miR-10a levels were compared between nonsurvivors (*n* = 17) and survivors (*n* = 45). (b) ROC curves present sensitivity and specificity of PBMC miR-10a, CRP, and PCT for predicting 28-day mortality.

All enrolled patients were monitored for 28 days after enrollment or until death. PBMC miR-10a levels at admission were significantly lower in nonsurvivors (*n* = 17) than in survivors (*n* = 45) (*p* < 0.05) (Figure 4(a)). The performance of miR-10a, PCT, and CRP for predicting 28-day mortality was evaluated by ROC curve analysis between hospital deaths and survivors for all enrolled patients. PCT performed the best for predicting 28-day mortality (AUC = 0.795; 95% CI, 0.664-0.920; *p* < 0.05). PBMC miR-10a (AUC = 0.699; 95% CI, 0.656-0.833; *p* < 0.05) displayed a similar prognostic performance as CRP (AUC = 0.705; 95% CI, 0.568-0.841; *p* < 0.001) (Figure 4(b)).

### 3.6. PBMC MAP3K7 Levels at Admission Are Higher in Sepsis Patients

**Figure 5:**
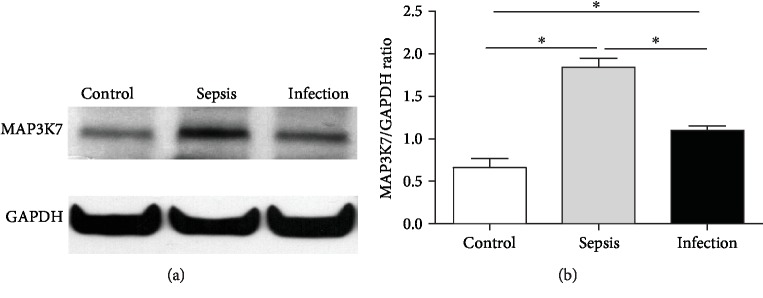
MAP3K7 expression in PBMC of sepsis patients with respect to infection and control. PBMC were collected from sepsis patients, infection patients, and healthy controls at admission. MAP3K7 protein expression in PBMC was examined via Western blot analysis. (a) A representative Western blot is shown. (b) Data are presented as mean ± SEM, *n* = 6. ^∗^*p* < 0.05.

It has been reported that miR-10a targets MAP3K7 and IRAK4, which act upstream of NF-*κ*B activation [22]. To determine whether altered miR-10a expression affected the level of MAP3K7 and IRAK4 in sepsis, PBMC from sepsis patients, infection patients, and healthy controls at admission were examined via Western blot analysis. Levels of MAP3K7 from sepsis patients were significantly higher than those of infection patients and healthy controls. In addition, MAP3K7 expression in infection patients was higher than that of control (Figures 5(a) and 5(b)). However, there was no difference in IRAK4 levels among the three groups (data not shown). These findings suggest that MAP3K7 is a target of miR-10a in immune cells.

### 3.7. Polymicrobial Sepsis Decreases the Levels of miR-10a in PBMC

**Figure 6:**
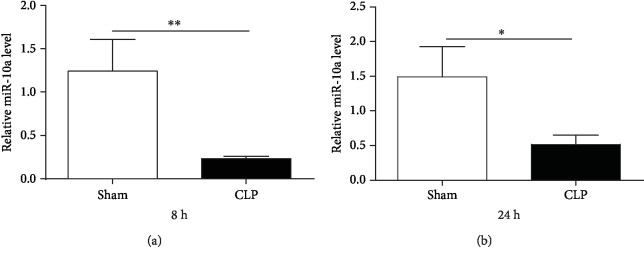
miR-10a expression in a mouse model of sepsis. PBMC were isolated from whole blood of C57BL/6 mice after 8 h (a) and 24 h (b) of sham or CLP surgery. miR-10a expression was determined via qRT-PCR. Data are presented as mean ± SEM, *n* = 6. ^∗^*p* < 0.05 and ^∗∗^*p* < 0.01.

To determine the effects of polymicrobial sepsis on the expression of miR-10a, sepsis was induced in a mouse model by CLP. PBMC were harvested at 8 h and 24 h after CLP and examined for miR-10a levels via qRT-PCR. As shown in Figure 6(a), polymicrobial sepsis significantly decreased the expression of miR-10a as early as 8 h after CLP surgery compared with sham operation (*p* < 0.01). PBMC miR-10a levels remained significantly diminished at 24 h after surgery (*p* < 0.05) (Figure 6(b)). These data demonstrate that miR-10a expression is reduced by polymicrobial sepsis and may serve as an early biomarker for sepsis.

### 3.8. miR-10a Overexpression Suppresses LPS-Induced Inflammatory Response In Vitro

**Figure 7:**
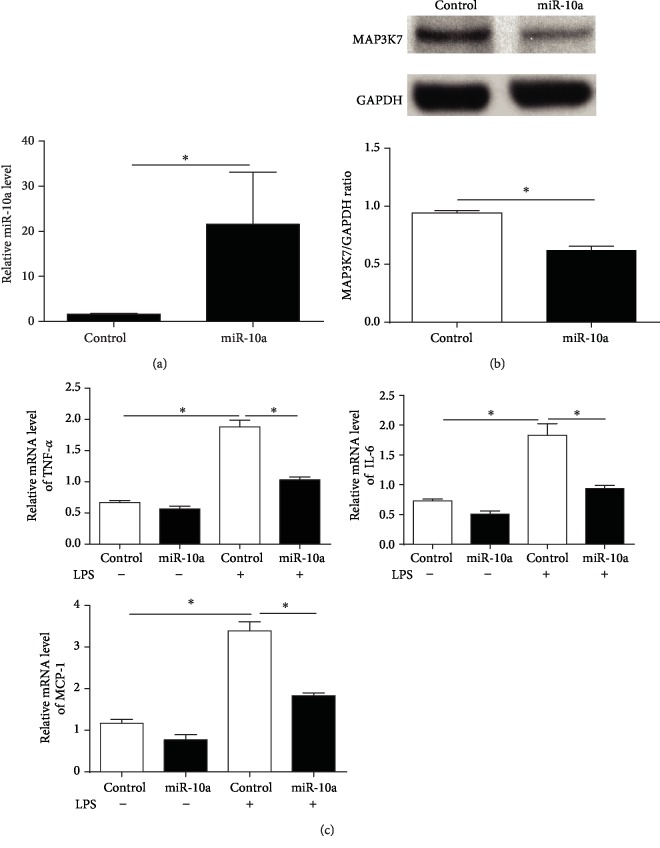
Effect of miR-10a overexpression on LPS-induced inflammatory cytokine production in monocytes. THP-1 monocytes were transduced with lentiviral miR-10a or miR-control. (a) Four days after the transduction, miR-10a levels in THP-1 cells were determined via qRT-PCR. (b) MAP3K7 expression in transduced THP-1 cells was determined via Western blot analysis. (c) The transduced cells were treated with or without LPS (100 ng/ml) for 24 h. Levels of IL-6, TNF-*α*, and MCP-1 in treated cells were assayed via qRT-PCR. Data are presented as mean ± SEM, *n* = 4. ^∗^*p* < 0.05.

We then asked whether overexpression of miR-10a could modulate LPS-induced inflammatory response in vitro. THP-1 monocytes expressed high levels of miR-10a after having been transduced with lentivirus expressing miR-10a (Figure 7(a)). miR-10a-transduced cells had lower expression of MAP3K7, further supporting that MAP3K7 is a target of miR-10a (Figure 7(b)). In addition, miR-10a overexpression suppressed the mRNA expression of IL-6, TNF-*α*, and MCP-1 induced by LPS as determined by qRT-PCR (Figure 7(c)). These data suggest that miR-10a is a negative regulator for proinflammatory cytokines induced by LPS.

## 4. Discussion

To the best of our knowledge, this is the first report demonstrating that PBMC miRNAs could serve as biomarkers for the diagnosis and prognosis of sepsis. Our results showed that sepsis patients presented significantly reduced expression of miR-10a compared with both infection patients and healthy controls. There was a significantly negative correlation between miR-10a levels and disease severity scores. In addition, PBMC miR-10a was at least as good as PCT and CRP in differentiating sepsis and infection. PBMC miR-10a levels were significantly lower in nonsurvivors than in survivors and predictive of 28-day mortality. Reduced miR-10a expression was also validated in a mouse model of polymicrobial sepsis. Furthermore, overexpression of miR-10a in THP-1 cells reduced the expression of MAP3K7 and proinflammatory cytokines induced by LPS, demonstrating the anti-inflammatory effect of miR-10a.

Sepsis-3 defines sepsis as life-threatening organ dysfunction resulting from dysregulated host response to infection. An inflammatory response which is part of a normal response to infection does not necessarily indicate sepsis. Early diagnosis of sepsis relies on laboratory biomarkers and clinical scores. However, most common biomarkers for sepsis diagnosis including PCT, CRP, and interleukin 6 have limited specificity and sensitivity. Our study demonstrated that levels of PBMC miR-10a at admission were significantly lower in sepsis patients than in infection patients and negatively associated with disease severity scores. In addition, PBMC miR-10a could distinguish sepsis from infection in our study population (AUC = 0.804; 95% CI, 0.688-0.920; *p* < 0.001). These findings provide evidences that PBMC miR-10a has the potential to be a novel diagnostic marker for sepsis. Furthermore, low PBMC miR-10a levels predicted an unfavorable short-term outcome, suggesting the role of PBMC miR-10a in the prognosis of sepsis. Given the complex pathophysiology of sepsis, no single biomarker of sepsis may be ideal for diagnosis. The application of multiple biomarkers has been reported to provide improved diagnostic accuracy to differentiate sepsis from infection [23]. Therefore, the combination of PBMC miR-10a with traditional biomarkers may be applied for the accurate diagnosis of sepsis.

During the past 10 years, circulating miRNAs have been explored as new biomarkers for sepsis. Analysis of miRNAs has the advantage of earlier detection compared to protein biomarkers since protein expression is located downstream of miRNA function. In addition, miRNA expression may enhance risk stratification and serve as a useful prognostic biomarker for sepsis. Such tool could provide guidance for early and aggressive intervention. However, circulating miRNAs are not directly related to cytokine-producing immune cells and may not be the earliest biomarkers for sepsis. Almost all cells exposed to serum are able to produce circulating miRNAs including neutrophils, platelets, lymphocytes, monocytes, endothelial cells, and endothelial progenitor cells [24, 25]. Secondly, a study in circulating miRNAs could not pinpoint the direct target proteins of the miRNAs in vivo and examine the detailed molecular mechanisms downstream of the miRNAs. Therefore, we evaluated the application of PBMC miRNAs as biomarkers for sepsis since PBMC are a major contributor of inflammatory cytokines during sepsis. Accumulating evidences support the application of PBMC miRNAs as disease biomarkers. Winger et al. reported that PBMC miRNA levels in the early first trimester predicted adverse outcomes such as miscarriage and late preeclampsia [26]. In treated HIV-1-infected individuals, Ballegaard et al. found that miR-210, miR-7, and miR-331 in PBMC were differentially expressed and correlated with biomarkers of systemic inflammation [27]. Dong et al. revealed that elevated expression of miR-24, miR-33a, miR-103a, and miR-122 in PBMC was associated with increased risk of coronary artery disease [28].

miR-10a has an anti-inflammatory effect and plays an essential role in the inflammatory response of immune cells [29]. Takahashi et al. documented that TGF-*β* and retinoic acid induced the expression of miR-10a, which constrained the differentiation of Th17 cells and inhibited the production of proinflammatory IL-17A [30]. Wu et al. discovered that miR-10a inhibited mucosal inflammatory response via targeting IL-12/IL-23p40 and NOD2 and blocking the immune response of Th1/Th17 cells [17]. Mu et al. showed that miR-10a levels were reduced in the fibroblast-like synoviocytes of rheumatoid arthritis patients compared with osteoarthritis controls [31]. It has been demonstrated that miR-10a could exert anti-inflammatory effects by targeting several members of the NF-*κ*B pathway. Njock et al. found that inhibition of miR-10a in monocytes abolished the inhibitory effects of endothelial cell-derived extracellular vesicles on proinflammatory genes via upregulating IRAK4 [32]. Fang et al. discovered that endothelial miR-10a levels were lower in the atherosusceptible regions than in the atheroresistant regions in the pig. The endothelium in atherosusceptible regions had upregulated the expression of MAP3K7 and BTRC as well as NF-*κ*B activation [22]. In addition, the induction of miRNA-10a via retinoic acid receptor-*α* or retinoid × receptor‐*α* agonists blocked the formation of an atherosclerotic lesion in rats [33]. In the present study, MAP3K7 levels in PBMC were elevated in sepsis patients and diminished in THP-1 cells overexpressing miR-10a, supporting that MAP3K7 is a target of miR-10a in immune cells.

Our study has several limitations. First, the sample size is small, although a small number of patients have been used in similar studies [34]. Further investigation is warranted to confirm the present findings. Our report is also limited by studying a single time point at admission in a disease with a prolonged course and complex cellular mechanisms. A time course study of PBMC miR-10a expression is warranted to confirm the utility of miR-10a as a biomarker for sepsis. Furthermore, it has been documented that miR-10a is expressed by all immune cell types in PBMC including T cells, B cells, natural killer cells, dendritic cells, and monocytes [17, 35]. This study is still incapable of concluding the cell type(s) contributing to the reduced expression of miR-10a in sepsis.

## 5. Conclusions

In summary, miR-10a has an anti-inflammatory capacity during immune response to sepsis. PBMC miR-10a levels correlate with the disease severity of sepsis. Importantly, low PBMC miR-10a expression is a diagnostic biomarker for sepsis and predictive of 28-day mortality. Due to the small sample size of the present study, a larger multicenter study will determine the value of a miR-10a-based biomarker for sepsis diagnosis and prognosis.

## Figures and Tables

**Table 1:** Demographic and clinical characteristics of sepsis patients.

Variable	Healthy controls (*n* = 20)	Infection (*n* = 21)	Sepsis (*n* = 41)	*p* value
Age (years)	65.6 ± 8.88	72.33 ± 15.59	74.49 ± 12.58	>0.05
Male/female	11/9	11/10	28/13	
SOFA score		0.48 ± 0.50	8.65 ± 3.41	<0.01
APACHE II score			27.78 ± 7.66	
Death (*n*)		0	17	
Microbiology of patients (*n*)				
Gram-positive		6	7	
Gram-negative		12	18	
No organism cultured		3	14	
Viral		0	2	
Type of infection (*n*)				
Pneumonia		21	31	
Severe cholangitis		0	2	
Other		0	8	
CRP (mg/l)		104.6 ± 85.52	160.69 ± 94.2	<0.05
PCT (mg/l)		2.71 ± 6.15	8.94 ± 9.77	<0.01

Data are presented as patient number or mean ± SD. APACHE II: Acute Physiology and Chronic Evaluation II; SOFA: Sequential Organ Failure Assessment; CRP: C-reactive protein; PCT: procalcitonin.

## Data Availability

The dataset supporting the conclusions of this article is included within the article.

## Abbreviations

PBMC: Peripheral blood mononuclear cells
CLP: Cecal ligation and puncture
MAP3K7: Mitogen-activated kinase kinase kinase 7
qRT-PCR: Real-time quantitative reverse transcriptase polymerase chain reaction
APACHE II: Acute Physiology and Chronic Evaluation II
SOFA: Sequential Organ Failure Assessment
CRP: C-reactive protein
PCT: Procalcitonin
ICU: Intensive care unit
ROC: Receiver operating characteristic
IQR: Interquartile range
AUC: Area under the curve.
